# Study on the law of surface subsidence in layered mining of thick coal seam with medium hard roof

**DOI:** 10.1038/s41598-023-42012-5

**Published:** 2023-09-07

**Authors:** Xiugang Liu, Fei Wei, Zhixiang Tan, Zaibing Jiang, Yi Wang, Jie Zhang

**Affiliations:** 1 China University of Mining and Technology, Beijing, 100083 China; 2 China Coal Research Institute, Beijing, 100013 China; 3 CCTEG Xi’an Research Institute (Group)Co, Ltd, Xi’an, 710077 China; 4 National Time Service Center, Chinese Academy of Science, Xi’an, 710600 China; 5 University of Chinese Academy of Science, Beijing, 100049 China; 6 JiangSu Key Laboratory of Resource and Environment Information Engineering, China University of Mining and Technology, Xuzhou, 221116 China

**Keywords:** Natural hazards, Geodynamics, Geomorphology, Geophysics, Petrology, Tectonics

## Abstract

In this study, the change law of the surface subsidence coefficient under the condition of thick coal seam layered mining was investigated. The study is based on the measured subsidence data of the 1210-working face of the Mengba mine surface mobile observation station after the first- and second-layer mining. UDEC numerical simulation software was used to simulate the variation of surface subsidence coefficient after the first, second, third, fourth, fifth-, and sixth-layer mining when the thickness of slicing mining is 5 m. The maximum relative error between the simulated result and the measured result of the subsidence coefficient *q* is 2.7%, which further verifies the correctness of the established model. Moreover, the simulation results show that with the increase of the cumulative mining thickness, the subsidence coefficient *q* of the surface presents a segmented characteristic. When the cumulative mining thickness does not reach 25 m, the subsidence coefficient of the surface gradually increases with the increase of the mining thickness. On the other hand, when the cumulative mining thickness reaches 25 m, the subsidence coefficient of the surface will tend to a constant value and no longer change with the increase of the mining thickness. Finally, the calculation formula between the surface subsidence coefficient and the cumulative mining thickness of layered mining under the condition of medium hard roof is fitted, which provides a parameter basis for coal seam mining with similar geological conditions.

## Introduction

The stratum and ground surface will move and deform due to the destruction during the primary coal mining. The underground strata movement and surface movement deformation will occur once more whenever mining its upper or lower coal seam again. This process is known as repeated mining^1^. Nowadays, given the large average recoverable thickness of thick coal seams, a fully mechanized top-coal caving mining method has been mainly adopted. Meanwhile, the large mining intensity of the fully mechanized caving will inevitably aggravate the damage to the rock seam above. The ground subsidence coefficient (*q)*, the main influence angle tangent (tanβ), and other angular parameters are different from those of the primary mining. At present, the research on the law of repeated mining of thick coal seams mainly focuses on the mining depth ranging from 3.5 to 15 m^2^, while few studies investigated mining depth of more than 15 m^3^. The slice mining of thick coal seams is popular, and the space above the mining area is larger than that of single-layer mining, and the movement range of the overlying rock layer is also increased. Therefore, the study on surface subsidence and defamation law is of high significance.

Yu et al.^4^ investigated the relationship between the development of the water suture zone in the overlying strata and different mining thicknesses, which revealed the relationship between the development height of the water suture zone and the mining thickness of the coal seam, with certain guiding significance. Li et al.^5^ applied the numerical simulation software UDEC to simulate and study the development height of “three zones” after the mining of thick coal seams, and the results showed that the sinking value of the overlying strata roof gradually decreased with the increase of the distance between the roof and the coal seam to a constant value. Wang et al.^6^ obtained the surface subsidence law and related rock movement parameters after mining of thick coal seams under comprehensive discharge conditions through the analysis of the actual measurement study of the surface movement observatory in the Baodian mine, which provided a certain basis for the mining of coal seams under similar geological mining conditions in China. The subsidence law of the surface caused by mining when the accumulative mining thickness is greater than 15 m is yet to be studied. Mastering the movement and deformation law of the surface of thick coal seam under the condition of stratified mining can provide the basis for the mining of coal seams in other mining areas under similar conditions in China. In this paper, taking the Barapukuli coal mine (referred to as Mengba mine) in Bangladesh as an example, a model was constructed by combining the measured subsidence data of surface station to simulate the law of the surface subsidence coefficient in the layered mining of thick coal seams, and based on the simulation results of the UDEC numerical simulation software. The simulation data and the actual measurement data were analyzed by regression to ensure the reliability and reasonableness of the simulation parameters, and the formula between the surface subsidence coefficient and the accumulative mining thickness under the layered mining conditions was fitted, which is expected to provide a parameter basis for the mining of similar coal mines in China.

## Overview of the mining area

The Mengba Mine is a modern large mine located in the northwest of Bangladesh with a design capacity of 1 million tons per year and a service life of 60 years. The mine covers 4.9 km in length from north to south and 0.3–1.9 km in length from east to west. The topography of the mine area is mainly made up of a plain formed by the alluvial deposits of the Ganges and Jamuna rivers, with a flat surface and a small slope, high in the north and low in the south. From the generalized geological section of the mine area, it can be observed that the mine area contains 7 layers from top to bottom, namely I, II, III, IV, v, VI, and VII coal seams, with a total thickness of about 75 m and a coal-bearing factor of 20%. The recoverable thickness of VI coal reaches 36 m, which is distributed in the whole area and is the main coal seam for mining. The direct roof of the coal seam is a medium-hard coarse-grained feldspar sandstone with a thickness of 95 m. Due to the large thickness of the coal seam, the method of slicing mining is adopted, and the mining thickness of a single layer is about 5 m. The first stratum was mined from 2005 to 2012, and mining of the second stratum of VI coal is currently underway.

## The establishment of the surface observation station

The 1210-mining face has an east–west full trend line of 1,200 m length and a north–south full incidence line of 500 m length. The former has 50 points and the latter 20 points. The thickness of single-layer mining is 5 m.

## Mathematical modeling

### Geometric modeling

**Figure 1:**
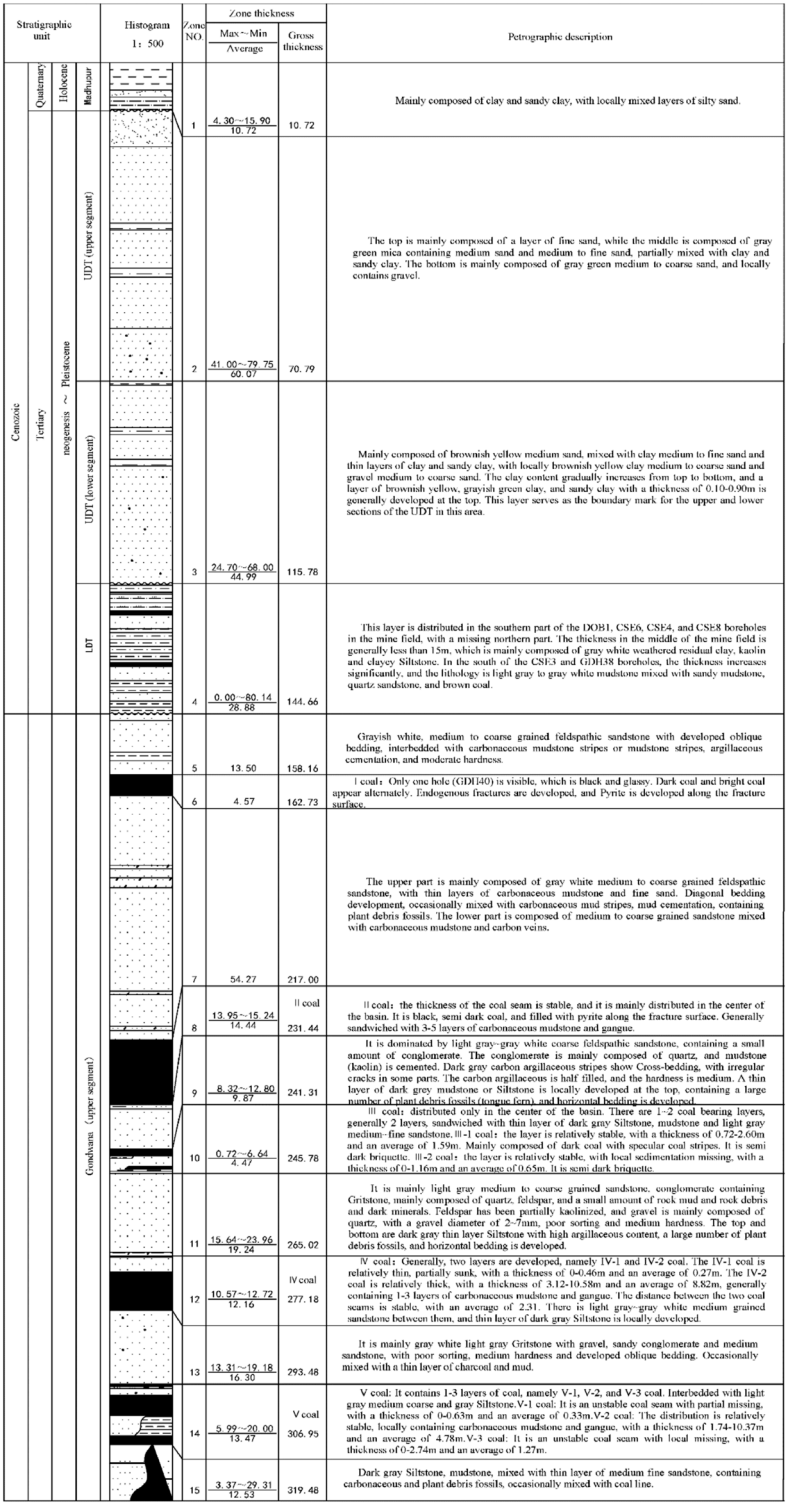

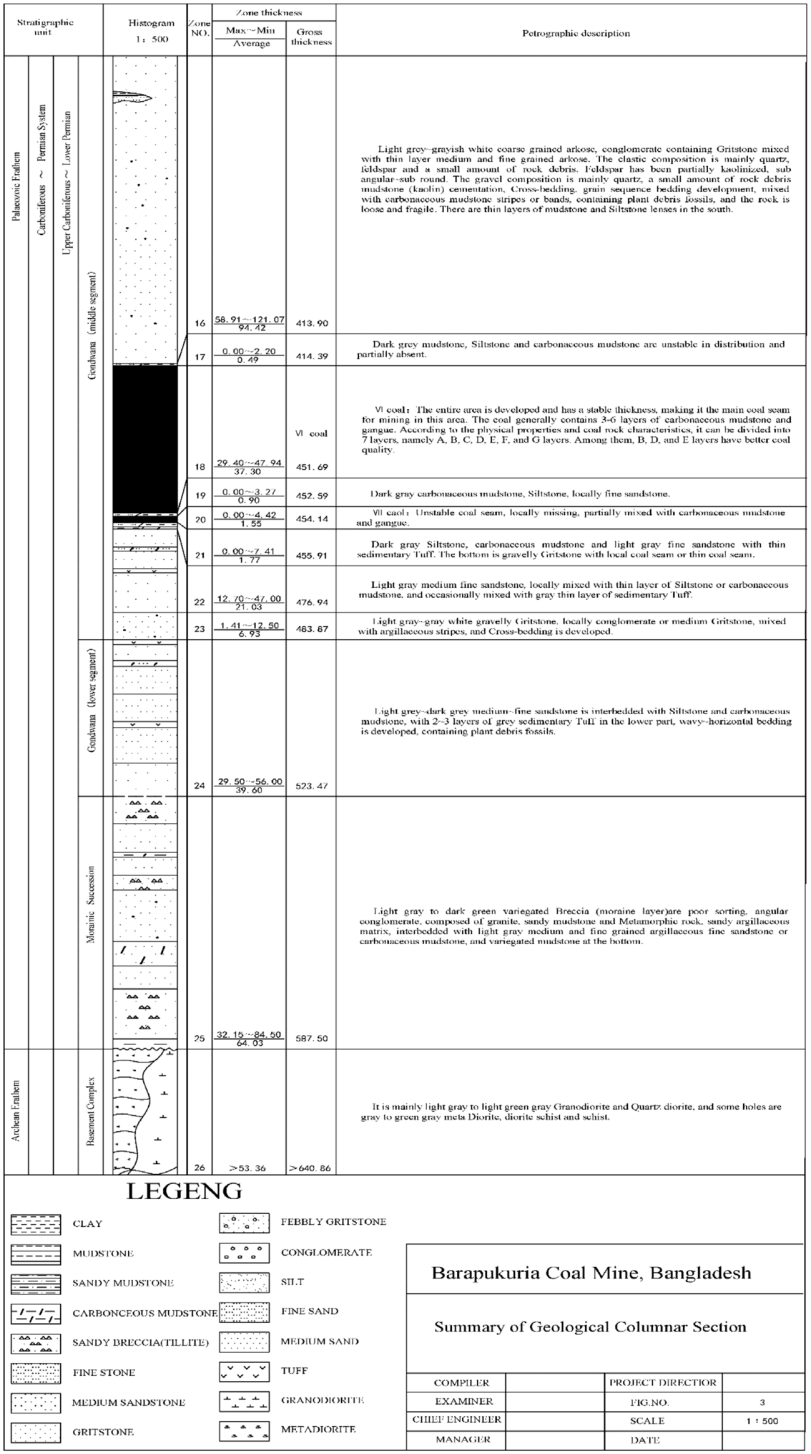
Simulation diagram.

This simulation of the movement changes of the surface after coal seam mining utilizes the numerical simulation software UDEC4.0 to model the geological mining conditions of the 1210-mining face in the Mengba mine area with the thickness of a single seam mining being 5 m. The actual strata with thin thickness and similar rock properties were combined together^7–11^ and treated as one layer to facilitate modeling during the numerical simulation. The overburden above the 1210-working face was divided into 17 parts after the combination. Where part 16 represents the Lower Dupi Tila (LDT) and part 17 is the Upper Dupi Tila (UDT), as shown in Fig. 1.

### Simulation of the mining recovery

This article mainly simulates the subsidence of the surface after layered mining of thick coal seams, and the numerical simulation adopts a one-time full-mining method. According to the mining plan of the 1210-working face, the first stratum was mined first, and the second, third, fourth, fifth, and sixth stratum will be mined successively after the various movement and deformation of the ground surface being balanced. To obtain the subsidence coefficient of the ground surface after fully mining the coal seam, the mining length along the trend direction is 600 m (1.5 times the average mining thickness), and a boundary of 400 m is taken on each side, so the total length of the model built is 1,400 m.

### Physical parameters of each rock formation

The Mohr–Coulomb yield criterion was used to determine the failure of the rock mass throughout the simulation, and plastic flow was not considered. The physical parameters of each rock layer of the numerical simulation model were determined according to the generalized geological section of the Mengba mine. The physical parameters of the main rock formations used in the simulation are shown in Table 1.

**Table 1:** Physical and mechanical parameters of each rock formation.

Mechanical	Density ρ/(kg/m3)	Elastic modulus E/(MPa)	Poisson's ratio μ	Internal friction angle/(°)	Cohesion/(MPa)	Tensile strength/(MPa)	Simulation layer thickness/(m)
Litholo							
Bottom plate	2,600	1 200	0.23	33	2.12	0.63	48
VI coal	1,430	751	0.16	20	3.20	0.6	36
Coarse-grained feldspar sandstone	2,500	220	0.21	31	2.00	0.25	95
Interbedded gray siltstone and mudstone	2,600	242	0.23	33	2.71	0.25	12
V coal	1,430	565	0.16	20	2.51	0.28	13
Gravelly coarse sandstone	2,680	336	0.22	37	3.00	0.25	16
IV coal	1,430	585	0.16	20	2.50	0.28	12
Coarse-grained sandstone	2,640	321	0.21	35	2.60	0.49	19
III coal	1,430	686	0.16	20	2.50	0.28	4
Coarse-grained feldspar sandstone	2,640	500	0.21	31	2.40	0.58	10
II Coal	1,430	569	0.16	20	2.50	0.28	14
Coarse-grained feldspar sandstone	2,640	775	0.23	35	2.50	0.35	54
I Coal	1,430	568	0.16	20	1.62	0.32	4
Coarse-grained feldspar sandstone	2,640	775	0.23	35	2.00	0.52	13
LDT	1,470	568	0.2	26	1.12	0.23	24
UDT	1,230	22	0.22	23	1.11	0.17	100
Topsoil	1,200	19	0.25	15	1.10	0.18	10

### Numerical simulation analysis

#### Analysis of overburden displacement and subsidence law

**Figure 2:**
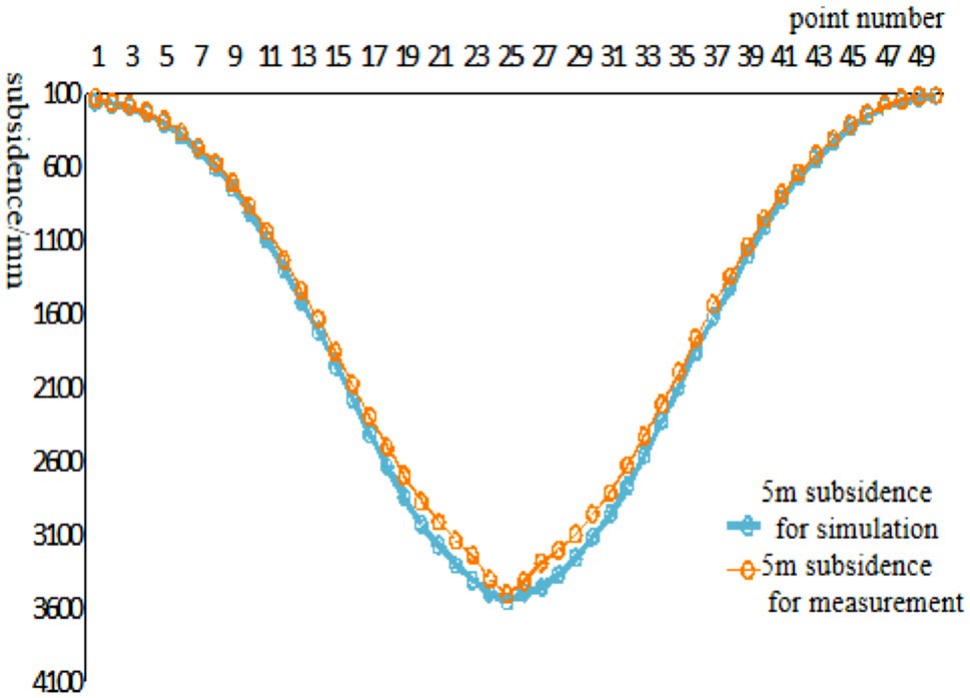
Comparison of measured data and simulated data.

Before analyzing the changes in surface subsidence coefficient and subsidence activation coefficient with the accumulative mining thickness, the correctness of the established model should be verified first, and the specific comparison results are shown in Fig. 2, Tables 2, and 3. The maximum sinking value of the ground was 3,550 mm after the recovery of the first stratum of the 1210-working face, and the numerical simulation results showed that the maximum sinking value of the ground was 3550 mm, corresponding to the mining thickness of 5 m. The sinking coefficient q = 0.71 was then obtained, and the sinking coefficient q = 0.70 was obtained by fitting the measured sinking data from the surface station using the referencing software. The maximum height of the hydraulic fracture zone after mining is 93.43 m, and the maximum development height of the water-conducting fractured zone after mining the coal seam was 96 m. The maximum relative error between the simulation results and the measured results q was 2.5%, and the maximum relative error of the water-conducting fractured zone was 2.7%. The maximum measured surface subsidence after the second stratification was 7,800 mm, and the value obtained from the numerical simulation was 7,900 mm, which resulted in a subsidence coefficient of q = 0.79. The subsidence coefficient q = 0.78 obtained by fitting the measured subsidence data from the surface station with the reference software was 1.2%, and the relative errors were less than the specified limit of 5%^12^. Therefore, the established simulation model of coal seam mining under specific geological mining conditions of Mengba Mine is correct.

**Table 2:** Comparison table of numerical simulation results.

Note:	Mining thickness/(m)	Maximum surface subsidence/(mm)	Sinking coefficient q
Measured data of the first layer	5	3500	0.70
Stimulated data of the first layer	5	3550	0.71
Measured data of the second layer	10	7800	0.78
Stimulated data of the second layer	10	7900	0.79

**Table 3:** Comparison table of results of numerical simulation of the water-conducting fracture zone.

Note:	Mining thickness/(m)	Development height of water-conducting fracture zone/(m)	Crack height mining thickness ratio
Measured data of the first layer	5	93.43	18.68
Stimulated data of the first layer	5	96.00	19.20

**Figure 3:**
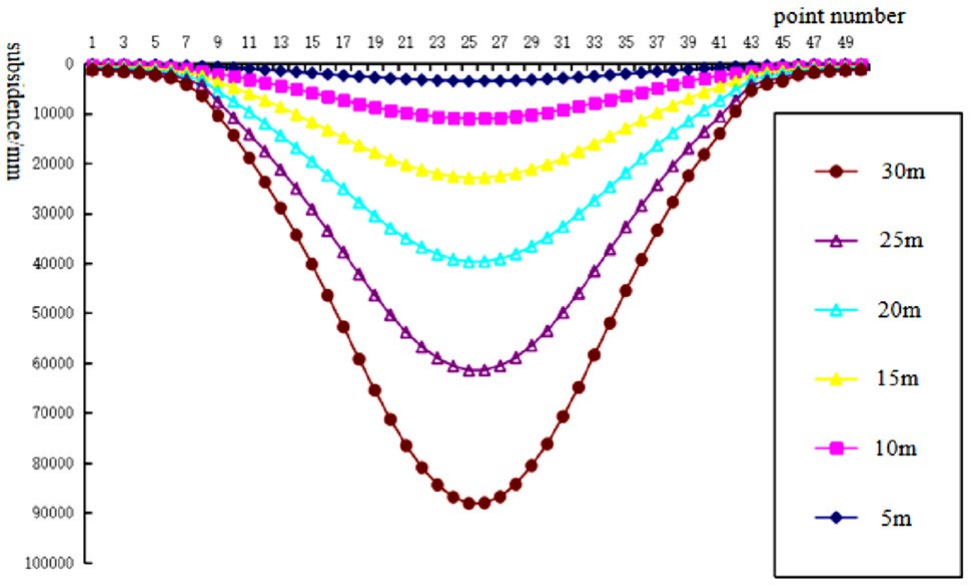
Simulating the subsidence curve of thickness change.

**Figure 4:**
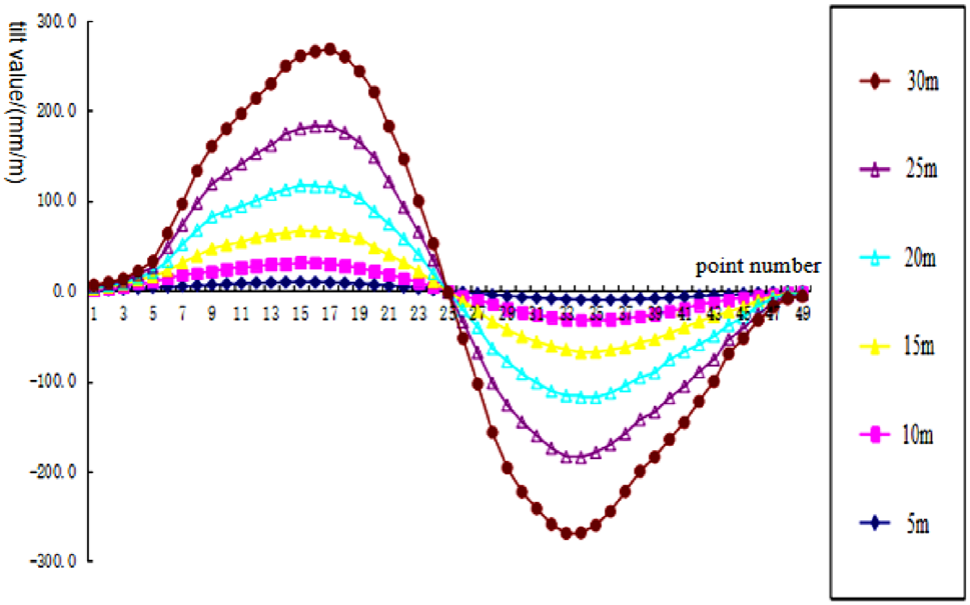
Simulating the tilt curve of thickness change.

**Figure 5:**
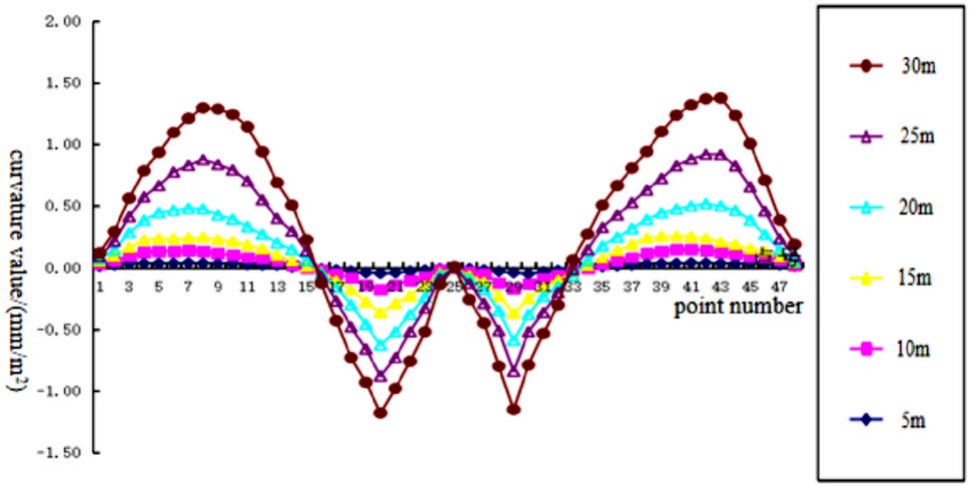
Simulating the curvature curve of thickness change.

**Figure 6:**
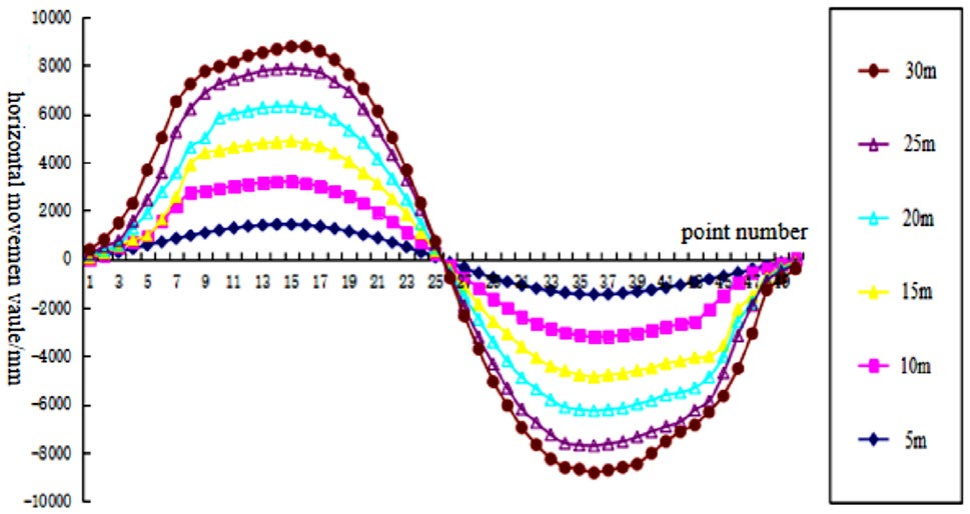
Simulating the horizontal movement curve of thickness change.

**Figure 7:**
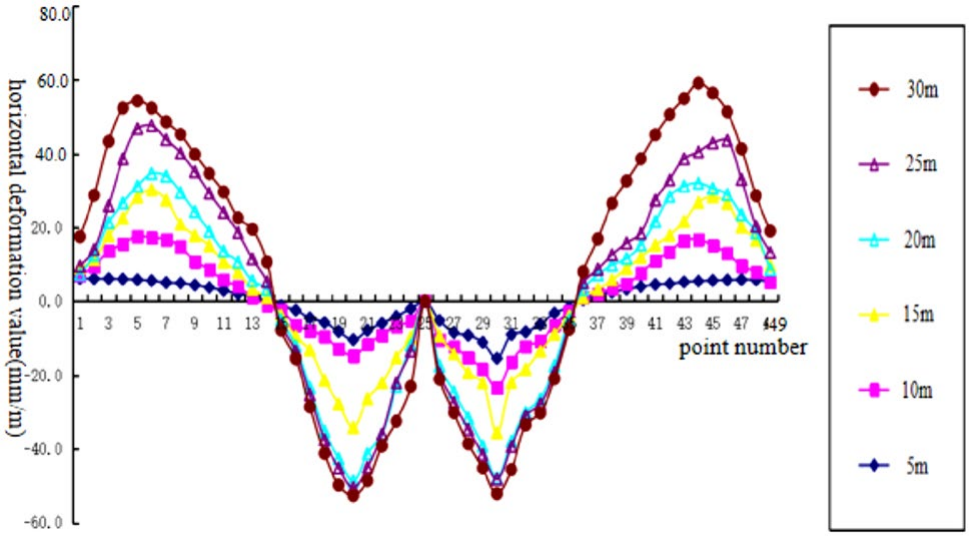
Simulating the horizontal deformation curve of thickness change.

To analyze the changes of surface subsidence law after the mining with the increase of cumulative mining thickness and the reliability of the numerical simulation, the cumulative mining thickness of 5 m, 10 m, 15 m, 20 m, 25 m, and 30 m were simulated under the same conditions as the actual mining of single seam, mining thickness, and mining method. The curves of surface subsidence, tilt, curvature, horizontal movement, and horizontal deformation with increasing cumulative mining thickness are shown in Figs. 3, 4, 5, 6, 7.

From the sinking curve simulating the change in mining thickness in Figs. 3, 4, 5, 6, 7, it can be seen that the surface sinking curve had flattened out after the coal seam recovery, indicating that the strike direction had reached the full mining level. There are two maximum points on the slope curve, a positive slope maximum point, and a negative slope maximum point, where the slope value of the mining center is zero. The curvature curve finally appears with three maximum curvatures; two positive maximum curvatures appear outward from the protected coal pillar; a negative maximum curvature appears between the protected coal pillar and the mining area; two maximum points of horizontal movement appear in the horizontal movement curve; three maximum points of deformation values appear in the horizontal deformation curve; a maximum negative point of horizontal deformation value appears between the protected coal pillar and the center of the mining area; a pair of maximum positive points of horizontal deformation value appear outward from the protected coal pillar, and the horizontal movement and horizontal deformation curves are similar to the tilt and curvature deformation curves. As the cumulative mining thickness increases, the values of surface subsidence, tilt, curvature, horizontal movement, and horizontal deformation all increase accordingly. In the sinking curve, the sinking value of the ground eventually reaches the maximum at the center of the mining area. The further away from the center of the mining area, the smaller the value of subsidence of the surface.

**Figure 8:**
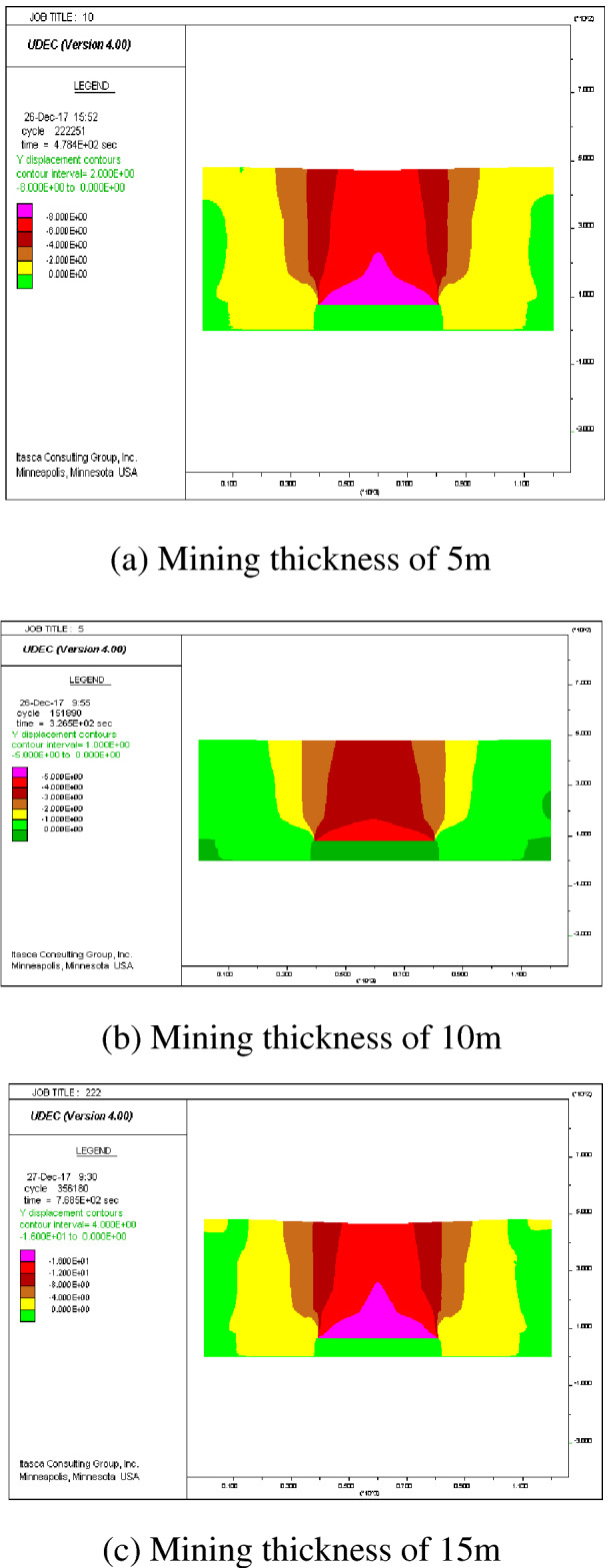

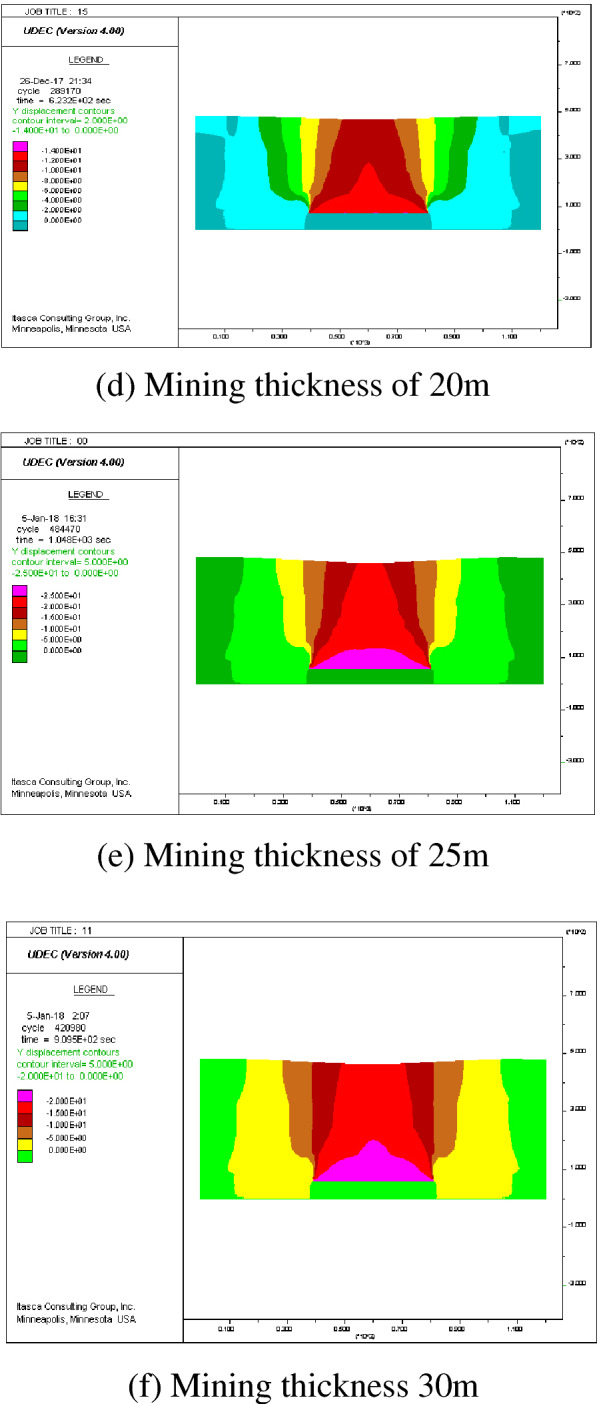
Displacement of overlying strata corresponding to different thicknesses.

After the mining, the coal seam can be divided from the center of the mining area upwards in order: caving zone, fractured zone, and bending subsidence zone. In this paper, the subsidence law of the ground surface after coal seam mining was investigated, so only the overburden deformation above the simulated excavation and the displacement of the ground surface in two directions, X and Y were extracted from the simulation results. Figure 8 shows the displacement clouds of overlying strata corresponding to different thicknesses. Afturer mining, the direct top plate was the first to collapse with the maximum movement and deformation value in the vertical direction. The further from the direct top plate, the smaller the deformation value of the overlying rock layer. Meanwhile, the deformation of the overlying rock layer and the sinking value of the ground gradually increases with the increase of the accumulative mining thickness, and the range of the central part of the ground surface sinking basin gradually increases as indicated by Fig. 8a,f. In the horizontal direction, the extent of the basin where the surface is affected by mining after coal seam mining increases gradually with the increase of cumulative mining thickness.

#### Result Analysis of subsidence coefficient

According to the numerical simulation results, the maximum surface subsidence and sinking coefficient of different total mining thicknesses are shown in Table 4.

**Table 4:** The maximum surface subsidence and sinking coefficient of different total mining thickness.

Cumulative mining thickness/(m)	Maximum surface subsidence/(mm)	Sinking coefficient q
5 m	− 3550	0.71
10 m	− 7900	0.79
15 m	− 12,600	0.84
20 m	− 17,800	0.89
25 m	− 23,000	0.92
30 m	− 27,600	0.92

For overlying rock formations with different properties, the activation coefficient for subsidence under each repeated mining condition can be calculated according to the following equation:

1 $$ q_{r1} = (1 + {\text{a}})q_{i} $$

2 $$ q_{{r{2}}} = (1 + {\text{a}})q_{{r{1}}} $$

where a is the repeated mining sinking activation coefficient,$q_{i}$,$q_{r1}$,$q_{r2}$ correspond to the corresponding sinking coefficients of initial mining, primary repeated mining, and secondary repeated mining, respectively.

According to the above equation, changes in the activation coefficient of subsidence during repeated mining are shown in Table 5.

**Table 5:** Changes in the activation coefficient of subsidence during repeated mining.

Lithology	Primary repeated mining	Secondary repeated mining	Tertiary repeated mining	Quaternary repeated mining	Fifth repeated mining and so on
Medium hard	0.11	0.06	0.06	0.03	0

**Figure 9:**
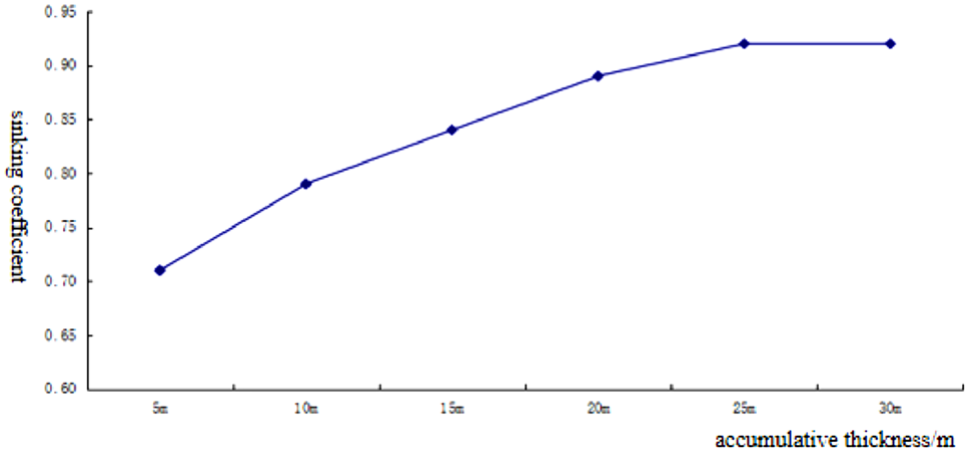
The relationship between the sinking coefficient and accumulative thickness.

According to the numerical simulation results in Tables 3 and 4, the following conclusions could be drawn:

(1) During the slicing mining of the thick coal seam, the sinking coefficient of the first repeated mining increased by 20% when the accumulative mining thickness was less than 15 m. The sinking coefficient of the secondary repeated mining increased by 10%, and the sinking coefficient of the subsequent repetitive mining no longer increased^13^. The cumulative mining thickness of the Mengba mine is 36 m, and the numerical simulation results showed that the surface subsidence coefficient was 0.79 after the secondary repeated mining, an increase of 11% relative to the primary repeated mining; the surface subsidence coefficient was 0.84 after the tertiary repeated mining, an increase of 7% relative to the secondary repeated mining; the surface subsidence coefficient was 0.89 after the quaternary repeated mining, an increase of 6% relative to the tertiary repeated mining; the surface subsidence coefficient was 0.92 after the fifth repeated mining, an increase of 3% relative to the quaternary repeated mining. After the cumulative mining thickness reached 25 m, q is intended to be a fixed value and keep constant. Furthermore, the repeated mining coefficients until the subsidence coefficient no longer increased after the fifth repeated mining.

(2) The activation coefficient a_1_ of sinking for primary repeated mining was 0.11, a_2_ for the secondary repeated mining was 0.06, a_3_ for the tertiary repeated mining was 0.06, and a_4_ for the quaternary repeated mining was 0.03. When the repeated mining time was more than five times, the activation coefficient of sinking no longer changed.

(3) The relationship between the surface subsidence coefficient and the cumulative thickness obtained by numerical analysis software is shown in Fig. 9:

The relationship between the surface subsidence coefficient and the cumulative thickness is as follows:

3 $$ {\text{q}} = {0}{\text{.0426}}\sum {{\text{m}} + {0}{\text{.696 }}} {\text{m}} \le {25} $$

4 $$ {\text{q}} = {0}{\text{.692 m}} > {25} $$

where $q$ is the subsidence coefficient,$\sum m$ is the cumulative thickness, m.

## Theoretical analysis

**Figure 10:**
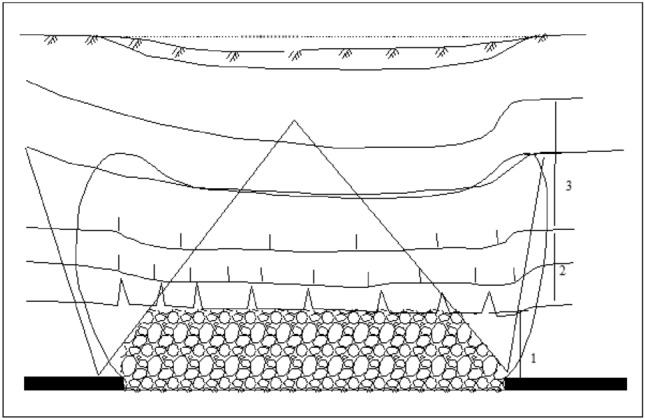
Schematic diagram of overburden failure. 1-caving zone; 2 fractured zone; 3-bending subsidence zone.

After the mining, the direct roof plate is subject to downward movement and deformation by gravity and the overburdened rock. Rock movement produces different forms of deformation in different areas, and according to the different forms of damage, the damaged rock layer above the goaf can be divided into three parts, as shown in Fig. 10.

In the caving zone where the rock layer collapses fully, the gap between the collapsed rocks increases and the volume expands. The volume of the rock after the span is larger than that before the collapse, and the collapsed rock layer fills the mining area.

The overlying rock layer above the caving zone produces cracks, departures, and fractures of a certain width, but the zone still maintains the original laminated structure, which is called the fractured zone. Water and sand can easily enter the mining area through the fracture zone, so when mining coal underwater, special attention should be paid to the development height of the fracture zone.

The bending zone refers to the area above the fracture zone to the surface, where the rock layers maintain a monolithic and laminated structure with good water barrier performance. The surface movement process in the bending zone is continuous and regular, and there are few off-layer cracks, which make it difficult for water and sand to pass through.

There are two main characteristics of slicing mining: the cumulative thickness of mining is large, and the surface is subject to repeated mining. After the destruction of the direct roof of the coal seam during the primary mining, the overlying rocks in the entire mining area are damaged and deformed, and the collapsed rocks of the overburdened rocks fill the mining void area, and the surface is deformed to a certain extent. The ruptured rocks of the lower coal seam damaged by secondary mining will cause the overburdened rocks to continue to collapse and fill the mining void area so that the gap between the rocks destroyed by the primary mining will be closed gradually, which increases the surface movement and deformation compared with the primary mining^14–18^. However, when the accumulated mining thickness reaches a certain amount, the gap inside the rock formation will be gradually compacted and will no longer increase with the mining operations. The number of repeated mining is related to the lithology of the overburdened rock on the roof. When the coal seam is repeatedly mined, there is a rock group with thickness separating the different layers between the mined coal seams, without disturbance during the primary mining, and the undisturbed rock layer plays a certain protective role for its upper layer during the repeated mining, thus making the repeated mining sinking coefficient gradually decrease with the repeated mining. When the coal seam is repeatedly mined, there is a rock group with thickness separating the different layers between the mined coal seams, without disturbance during the primary mining, and the undisturbed rock layer plays a certain protective role for its upper layer during the repeated mining, thus making the repeated mining sinking coefficient gradually decrease with the repeated mining. When the mining thickness reaches a certain value, all layers encounter destructive deformation, the protective effect gradually weakens or disappears^19–23^, and the repeated mining sink coefficient will gradually tend to zero and no longer change. According to the numerical simulation results, the subsidence coefficient of the ground gradually increased after the second, third, and fourth strata mining compared with the previous layer, and when the accumulated mining thickness reached 25 m, the subsidence coefficient of the ground surface tended to a constant value of 0.92. The experimental results are the same as the theoretical analysis.

## Conclusion

1. The subsidence coefficient q of the surface exhibits segmented characteristics as the cumulative mining thickness increases. Numerical simulation results show that the surface subsidence coefficient was 0.79 after the secondary slicing repeated mining, an increase of 11% than that after the primary slicing repeated mining; the surface subsidence coefficient was 0.84 after the tertiary slicing repeated mining, an increase of 7% than that after the secondary slicing repeated mining; the subsidence coefficient was 0.89 after the quaternary slicing repeated mining, an increase of 6% than that after the tertiary slicing repeated mining; the surface subsidence coefficient was 0.92 after the fifth slicing repeated mining, an increase of 3% than that after the quaternary slicing repeated mining. After the cumulative mining thickness reached 25 m, q is intended to be a fixed value and keep constant. And the repeated mining coefficients until the subsidence coefficient no longer increased after the fifth repeated mining. The activation coefficient a1 of sinking for primary repeated mining was 0.11, a2 for the secondary repeated mining was 0.06, a3 for the tertiary repeated mining was 0.06, and a4 for the quaternary repeated mining was 0.03. When the repeated mining time was more than five times, the activation coefficient of sinking no longer changed.
2. The calculation formula between the ground subsidence coefficient and cumulative mining thickness of layered mining under the condition of the medium-hard roof was fitted, which is expected to provide a parameter basis for the mining of coal seams in similar mines.

## Data Availability

The datasets used and/or analysed during the current study available from the corresponding author on reasonable request.
